# Phage Cocktail Alleviates Bacterial Canker of Kiwifruit by Modulating Bacterial Community Structure in Field Trial

**DOI:** 10.3390/microorganisms13010104

**Published:** 2025-01-07

**Authors:** Ran Hu, Xiaohan Xu, Yajun Jia, Cancan Zhu, Lin Wang, Minxin Song, Qian Xu, Mian Xia, Xiaoqing He, Yi Jin

**Affiliations:** 1 College of Biological Sciences and Biotechnology, Beijing Forestry University, Beijing 100083, China; ran.hu@ganlee.com (R.H.); xuxiaohan@bjfu.edu.cn (X.X.);; 2 Beijing Key Laboratory of Food Processing and Safety in Forestry, Beijing Forestry University, Beijing 100083, China; 3 Hainan Yazhou Bay Seed Laboratory, Yazhou 572024, China

**Keywords:** phage biocontrol, *Pseudomonas syringae* pv. *actinidiae*, endophytic microbiome, community assembly processes

## Abstract

Bacterial canker of kiwifruit is the most destructive bacterial disease caused by *Pseudomonas syringae* pv. *actinidiae*. Bacteriophages are regarded as promising biocontrol agents against kiwifruit bacterial pathogens due to their exceptional host specificity and environmentally friendly nature. However, the underlying mechanism of phages in the control of kiwifruit bacterial canker disease remains elusive. In this study, the field trial results showed that phage cocktail could significantly reduce the incidence of bacterial canker in kiwifruit. The high throughput sequencing results showed that the phage cocktail regulated the impact of pathogen invasion on branch endophytic communities, adjusted the diversity of the bacterial community structure, regulated the composition of rare taxa and abundant taxa, and increased the proportion of deterministic processes in community assembly processes. The phage cocktail significantly reduced the relative abundance of *Pseudomonadaceae*, *Pectobacteriaceae*, and *Yersiniacea*. Furthermore, the application of the phage cocktail resulted in an increase in the relative abundance of *Beijerinckiaceae*, *Sphingomonadaceae*, and *Xanthomonadaceae*, most of which are abundant taxa of the corresponding microbial communities. Additionally, the composition of rare taxa was also altered under the influence of phages. These findings offer perspectives on the phage-mediated biocontrol of kiwifruit bacterial canker and provide practical backing for the implementation of phage cocktails in sustainable agriculture.

## 1. Introduction

The flavor and nutritional benefits of kiwifruit have earned it the title of “the king of fruits” and contributed to its favorable reputation [1]. The bacterial canker caused by *Pseudomonas syringae* pv. *actinidiae* has been widely recognized as the most devastating disease in kiwifruit production. In the 1980s, *P. syringae* pv. *actinidiae* was first isolated and confirmed in Japan as the causative agent of bacterial ulcer disease in kiwifruits [2]. Subsequently, *P. syringae* pv. *actinidiae* was isolated and identified as the causal agent of kiwifruit canker disease in other countries [3]. Due to differences in certain biochemical characteristics and pathogenicity, etc., of *P. syringae* pv. *actinidiae*, the current *P. syringae* pv. *actinidiae* can be divided into six biovars [4]. The disease has caused significant losses in kiwifruit-growing countries, as reported by various sources [5,6,7,8]. Symptoms of the disease encompass crimson cracks on branches, the browning of fruits, and cankers accompanied by exudates on trunks and branches [9,10]. Compared to traditional pesticides, the application of bacterial inoculants for modulating the composition of plant rhizosphere microbiota is considered a more reliable strategy for disease management. In addition to their direct effects on pathogenic microorganisms, various beneficial rhizosphere microbes can enhance the defense mechanisms of the above-ground portions of plants, thereby reducing the incidence of diseases [11]. Concurrently, biocontrol agents can augment the abundance of beneficial bacteria within phyllosphere microbial communities and mitigate disease by displacing pathogenic bacterial niches [12,13]. Phage biocontrol utilizing plant pathogen-specific viruses to manipulate phyllosphere microbiota presents a potentially effective approach for safeguarding plants against diseases.

Bacteriophage refers to a group of bacterial viruses that can specifically lyse host bacteria, but which are not toxic to untargeted bacteria, animals, or plants [14]. In order to overcome the development of phage resistance and prolong therapeutic efficacy, phage cocktail therapy involving simultaneous administration of two or more types of phages is employed in clinical and agricultural disease management [15]. In studies focused on kiwifruit bacterial canker, various phages have demonstrated efficacy. In greenhouse experiments, a phage cocktail effectively reduced *P. syringae* pv. *actinidiae* load and mitigated leaf damage [16]. Phage PPPL-1 prevented bacterial canker development in kiwifruit [17]. Furthermore, a four-phage cocktail significantly reduced *P. syringae* pv. *actinidiae* in kiwifruit seedlings, laying a foundation for biological control strategies against kiwifruit bacterial canker [18]. According to our previous study, the four phages (PKb2b, PHB10b, PKb5a, and PHR10a) and the phage cocktail derived from them showed remarkable biological control efficacy both in vitro and in vivo [18]. Due to their non-toxicity towards plants and beneficial microflora, high selectivity for host bacteria, environmental safety, and ability to eradicate antibiotic-resistant bacteria, phages are considered environmentally favorable alternatives for the management of *P. syringae* pv. *actinidiae* infection in kiwifruit plants. However, limited research has been conducted on the impact of phages on branch endophytic microbes under field conditions.

Studies have demonstrated that the influence of bacteriophages can significantly alter the diversity and composition of phyllosphere bacterial communities. By reducing pathogen density, bacteriophages create niche spaces that facilitate the proliferation of beneficial bacteria [19]. It has also been demonstrated that various resident bacterial taxa can collaborate with applied bacteriophages to mitigate diseases. Furthermore, distinct rhizosphere or phyllosphere microbiomes may harbor functionally redundant bacteria, even though they belong to different taxa groups. Nevertheless, these bacteria have the capacity to inhibit the spread of pathogens and increase in abundance in response to phage application, jointly preventing further disease progression [11,20]. The microbial communities exhibit a remarkable level of diversity, characterized by the coexistence of numerous rare taxa alongside a limited number of abundant taxa [21]. According to previous studies, both abundant and rare taxa exhibit significantly distinct characteristics and functional traits across various ecosystems [22]. Abundant and rare taxa interact with each other, thus developing complex networks [23]. The report indicates that rare taxa play a positive role in their interactions with plants and feedback on plant diseases by enhancing the resilience of microbial communities [24]. It has been commonly accepted that stochastic and deterministic processes have an impact on the patterns of microbial community assembly [25]. The neutral theory posits that stochastic processes are governed by random factors, and it explains these processes by assuming a random equilibrium between the loss and gain of the microbial community [26]. The null model was applied to quantify various underlying mechanisms of the assembly process [27]. The contribution of the two processes that drive community assembly remain a subject of controversy. In light of this, it is imperative to investigate the modifications in community assembly processes under the influence of a phage cocktail. In this investigation, a combination of four highly efficient *P. syringae* pv. *actinidiae* phages, PKb2b, PKb5a, PHB10b, and PHB09, was utilized to formulate a phage cocktail, and its efficacy in field settings was examined. The findings elucidated the regulatory mechanisms of phage cocktail on the bacterial community structure and promoted the transition of the microbial community from a diseased state to a healthy state, thereby advancing research on biological control strategies for kiwifruit canker and providing essential theoretical groundwork and practical support for implementing phage biocontrol against this plant’s bacterial diseases.

## 2. Materials and Methods

### 2.1. Preparation of Phage Cocktail

Pathogenic *P. syringae* pv. *actinidia* strains (PsaJS1, PsaJS2, and PsaJS3) were isolated from canker in branches of kiwifruit from three neighboring orchards in Yongshun county, Jishou City, Hunan Province, and their pathogenicity has been confirmed in vivo. Biovar specific primers hopZ5-F2 and hopZ5-R2 [28] were used to identify the three pathogens as *P. syringae* pv. *actinidiae* biovar3. The host range of candidate phages was investigated by infecting different strains of *P. syringae* pv. *actinidiae* using the spot assay method [29] with PsaJS1, PsaJS2, PsaJS3, and PsaBJ530 (CGMCC1.19157) as the host strains. The bacterial sensitivity to a phage was determined based on the formation of a lysis-cleared zone surrounding the spot. Four *P. syringae* pv. *actinidiae* phage strains (PKb2b, PKb5a, PHB10b, and PHB09) previously isolated from kiwifruit branches, soil, or wastewater in our studies [18,30] exhibited effective bacterial lysis against all host strains and were utilized for the preparation of the phage cocktail.

The PsaBJ530 strain, previously isolated from canker-infected branches, served as the host for phage propagation. This strain was routinely cultivated in trypticase soy broth (TSB, Oxoid, Basingstoke, UK) medium at 25 °C until reaching an optical density of approximately 0.4 (OD_600_). To prepare the initial phage stocks, each phage was individually cultured with PsaBJ530 in TSB medium, followed by centrifugation and filtration steps using a 0.22 μm filter to isolate and purify the phages from bacteria. The supernatant was diluted and mixed with PsaBJ530, and the titer was determined using the double-layer agar technique. Four phages (10^10^ PFU/mL) were mixed evenly at 1:1:1:1 (*v*/*v*) to make a phage cocktail and diluted 100-fold before the field spray.

### 2.2. Field Trial Design

**Figure 1:**
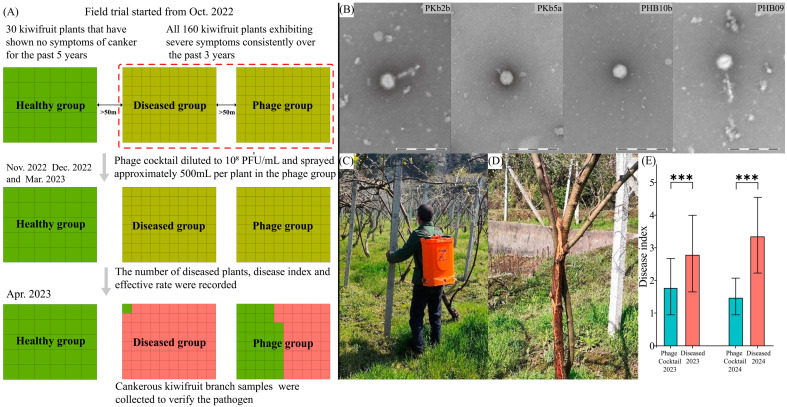
The morphology characteristics of four phages and the field trial. (**A**) The procedure of the field trial. Kiwifruit plants exhibiting canker symptoms are denoted by pink squares, while green squares represent asymptomatic plants. Green square: healthy plants. Khaki square: plants have a history of canker disease. Red square: diseased plants. (**B**) Transmission electron microscopy images of the phages PKb2b, PKb5a, PHB10b, and PHB09. Scale bars represent 200 nm. (**C**) Phage cocktail was sprayed in the field. (**D**) Cankers with exudates on *P. syringae* pv. *actinidiae*-infected kiwifruit branches. (**E**) Effects of phage cocktail on bacterial canker of kiwifruit disease index in field. Asterisks indicated significant differences between groups, as indicated by the *t*-test or Wilcoxon test (*** *p* < 0.001).

The field trial was performed in a kiwifruit orchard located in Jishou City (Hunan Province), with more-than-10-year-old plants of “Miliang-1” kiwifruits (*Actinidia deliciosa* cv. Miliang-1). This field trial was conducted from October 2022 to April 2023 and repeated from October 2023 to April 2024. The trial consisted of three groups: the healthy group, the diseased group, and the phage cocktail treatment group (phage group), each separated by a distance of over 50 m. All the plants were dispersed in various areas of the orchard and selected in accordance with the following rules: the healthy group comprised 30 kiwifruit plants that have remained asymptomatic for bacterial canker for the past five years, whereas both the diseased and phage groups included 80 kiwifruit plants, respectively, all exhibiting severe symptoms consistently over the last three years. Therefore, phage cocktail (diluted to 10^8^ PFU/mL, 500 mL of each plant) was sprayed on kiwifruit branches in mid-November during defoliation, December after pruning, and early March of the following year, respectively (Figure 1A). In the following April, the number of diseased plants, disease index, and effective rate were recorded. Effective rate = (number of cases in diseased group − number of cases in phage group)/number of cases in diseased group. The severity of bacterial canker disease (disease index) was recorded as the proportion of diseased branches or the proportion of main stem spots to stem girth using a scale ranging from 0 to 5 (0, no signs of bacterial canker; 1, the proportion value was less than 1/3; 2, the proportion value was more than 1/3 and less than 1/2; 3, the proportion value was more than 1/2 and less than 2/3; 4, the proportion value was more than 2/3; and 5, the kiwifruit plant was dead).

Five cankerous kiwifruit branch samples from the phage and diseased groups were collected, respectively, to verify the pathogen. The total bacterial DNA of the branch sample was extracted using the Super Plant Genomic DNA Kit (Polysaccharides and Polyphenolics-rich) (TIANGEN Biotech, Beijing, China). *P. syringae* pv. *actinidiae* universal PCR primers PsaF and PsaR [31] were used for pathogen detection. The experiment was conducted in triplicate for each sample.

### 2.3. Sample Collection

In April of 2023, five healthy plant branch samples were collected from the healthy group, while five canker branch samples were obtained from the diseased group. Additionally, in the phage treatment group, five asymptomatic and five diseased branch samples were collected. The collected branches were carefully placed in sterile plastic bags for transportation to the laboratory and subsequently stored at −80 °C until DNA extraction.

### 2.4. DNA Extraction and Sequencing

For branch endophytic microorganism investigation, the aforementioned frozen branches were surface sterilized with 75% ethanol for 1 min. Then, they were rinsed with sterile water three times. After snap freezing with liquid nitrogen and crushing to a powder with a sterile mortar and pestle, total genomic DNA samples were extracted using the Super Plant Genomic DNA Kit (Polysaccharides and Polyphenolics-rich) (TIANGEN Biotech, Beijing, China). The V3-4 hypervariable region of bacterial 16S rRNA gene was amplified with the universal primers 338F and 806R [32]. The PCR was carried out on a Mastercycler Gradient (Eppendorf, Hamburg, Germany) using 25 μL reaction volumes, containing 12.5 μL 2× Taq PCR MasterMix (Vazyme Biotech Co., Ltd., Nanjing, China), 3 μL BSA (2 ng/μL), 1 μL Forward Primer (5 μM), 1 μL Reverse Primer (5 μM), 2 μL template DNA, and 5.5 μL ddH_2_O. Cycling parameters were 95 °C for 5 min, followed by 28 cycles of 95 °C for 45 s, 55 °C for 50 s, and 72 °C for 45 s, with a final extension at 72 °C for 10 min. The PCR products were purified using an Agencourt AMPure XP Kit (Beckman Coulter, Inc., Indianapolis, IN, USA).

Deep amplicon sequencing was performed on Miseq PE300 platform at Allwegene Company (Beijing, China). After the run, image analysis, base calling, and error estimation were performed using Illumina Analysis Pipeline Version 2.6. The raw data underwent screening, removing sequences shorter than 120 bp and with quality scores ≤ 20, and containing ambiguous bases, mismatching primer sequences, and barcode tags. Qualified tags were denoised into ASVs using the unoise3 algorithm from Usearch (v10.0.240) software [33]. The BLAST tool (v2.14.0) was used to classify all sequences into different taxonomic groups against the SILVA138 database [34].

### 2.5. Bioinformatics Analysis

Sequence data analysis was mainly performed using QIIME2 and the R package (v4.3.1). Each group contained five replicates from five different kiwifruit plants. Alpha diversity and taxonomical differences were evaluated at various stages using non-parametric statistical tests (Kruskal–Wallis test). Based on Bray–Curtis distances, the beta diversity of the microbial community structure was compared by principal coordinate analysis (PCoA), and ANOSIM (Analysis of Similarities) was performed to assess the significant differences in microbial community structure between groups [35]. The branch endophytic microbial community co-occurrence network was calculated using the R package “picante” [36]. Subsequently, network visualization was implemented with Gephi (0.10.1). Additionally, using the “igraph” package and R version 4.3.1, we calculated the network topology and node-level topological properties. The relative abundance thresholds of 0.5% for abundant taxa and 0.01% for rare taxa were established as described [37]. The bacterial communities were classified into three distinct taxonomy categories; those falling in the middle were referred to as intermediary taxa [37]. To investigate the significance of deterministic and stochastic processes in generating the bacterial community structure, the null model and neutral community model were combined [38,39]. To infer the ecological processes in particular communities, we calculated the βNTI using the R packages “picante”, “vegan”, and R version 4.3.1 to quantify the deviation between the distribution of the βMNTD values of the observation and the βMNTD values of the null model [40]. Bray–Curtis-based taxonomic diversity is measured by the Raup–Crick (RCbray) metric. Community assembly is dominated by stochastic processes, as shown by |βNTI| < 2. Variable selection governs community assembly, according to βNTI > 2, and homogeneous selection predominates in this process, according to βNTI < 2. We combined βNTI (2) with RCbray (0.95) to determine the mechanisms of community assembly processes, such as heterogeneous selection, homogeneous selection, dispersal limitation, homogeneous dispersal, and undominated processes [41]. A random forest model was calculated using the R package “randomForest” to select important bacteria that correlated with the group.

### 2.6. Statistical Analysis

Disease index analysis was performed using GraphPad Prism 8.0 (GraphPad Software, San Diego, CA, USA). *t*-tests or Wilcoxon tests were used to determine differences between groups in disease index analysis, alpha diversity analysis, topological features of co-occurrence, and comparison of βNTI for kiwifruit branch endophytic bacterial communities. The *t*-test was used when the data met the necessary assumptions. Otherwise, Wilcoxon tests or PERMANOVA was used to test for differences between groups. Statistical significance was established at *p* < 0.01. *, **, and *** represented *p* < 0.05, *p* < 0.01, and *p* < 0.001, respectively. Lowercase letters in the figures indicated significant differences at *p* < 0.05, based on the *t*-test or Wilcoxon test.

## 3. Results

### 3.1. Biocontrol Effect of Phage Cocktail Against Kiwifruit Bacterial Canker in Field Trial

The field trial process was shown in Figure 1A. Four lytic *P. syringae* pv. *actinidiae* phages (PKb2b, PKb5a, PHB10b, and PHB09) (Figure 1B) were selected to formulate the phage cocktail. A field trial was conducted to investigate the biocontrol efficacy of the phage cocktail spray against kiwifruit canker (Figure 1C). In the healthy group, all 30 kiwifruit plants exhibited no symptoms of canker for two consecutive years. In the diseased group of 2023, typical symptoms of bacterial canker disease were observed in 79 out of 80 kiwifruit plants, and 75 plants in the diseased group of 2024 showed canker symptoms (Table 1) (Figure 1D). Importantly, only 42 (2023) or 38 (2024) out of the 80 kiwifruit plants in the phage cocktail treatment group showed cankerous symptoms (Table 1). The efficacy rate of the phage cocktail treatment was calculated to be 46.8% in 2023 and 50.7% in 2024. Upon evaluating the severity of the bacterial canker disease, the mean disease index for the phage cocktail group was significantly lower than that for the diseased group in both 2023 and 2024 (Table 1) (Figure 1E). Furthermore, pathogen *P. syringae* pv. *actinidiae* was detected in all 10 symptomatic branch samples collected from both phage-treated and untreated diseased groups, providing evidence that bacterial canker in the field was caused by *P. syringae* pv. *actinidiae*. In summary, compared to the diseased group, treatment with the phage cocktail substantially reduced both the incidence rate and disease index of affected plants in the field.

### 3.2. Effects of Phage Cocktail on the Branch Endophytic Bacterial Community Structure

To elucidate the underlying mechanism of phage cocktail in controlling bacterial kiwifruit canker disease during the field trial, the phage cocktail treatment group was further divided into two distinct subgroups: the phage asymptomatic group and the phage diseased group.

**Figure 2:**
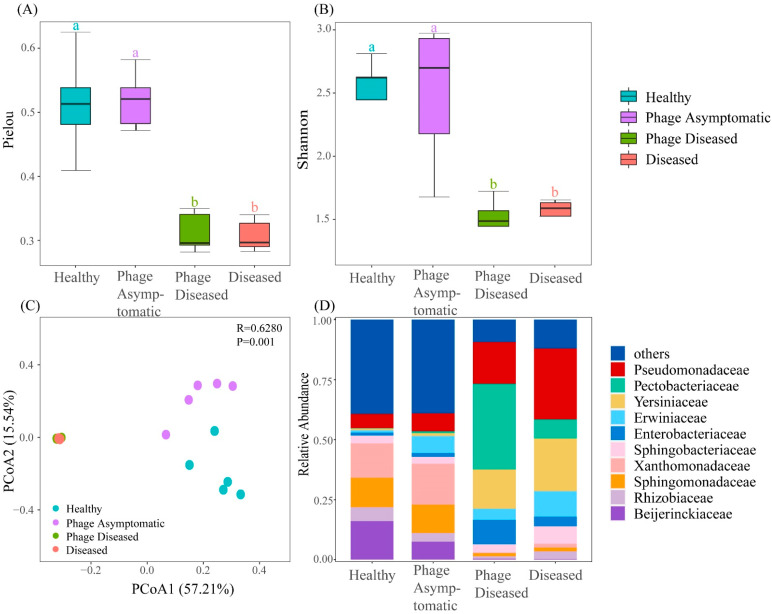
Diversity, composition, and community structure of branch endophytic bacterial community of kiwifruit. Pielou index (**A**) and Shannon (**B**) index of subcommunities. Means not sharing the same letter are significantly different by the *t*-test or Wilcoxon test (*p* < 0.05). (**C**) Principal co-ordinates analysis (PCoA) based on Bray–Curtis dissimilarities demonstrated the distance of the four groups, the first and second ordination axis accounted for 57.21% and 15.54% variations, respectively. (**D**) Taxonomic composition and relative abundance of the branch endophytic microbial community in kiwifruit branches. The families ranked outside the top 10 were grouped into “Others”.

The 16S rRNA gene sequencing statistics and ASV details are shown in Supplementary Tables S1 and S2. The alpha diversity index serves as a quantification of species richness. In the branch endophytic microbial communities, the Pielou index of both the healthy group and the phage asymptomatic group exhibited significant increases compared to the diseased and phage diseased groups (Figure 2A). Additionally, there was a notable decrease in the Shannon index for both the diseased group and the phage diseased group when compared to the healthy group and phage asymptomatic group (Figure 2B). The alpha diversity index shows no significant difference between the healthy group and the phage asymptomatic group, nor between the diseased group and the phage diseased group. These findings suggest that the healthy group and the phage asymptomatic group exhibited similar levels of bacterial community diversity and abundance, which were higher compared to those observed in the diseased group and the phage diseased group.

PCoA analysis was performed to analyze endophytic microbial communities in the kiwifruit branches. The horizontal axis accounted for 57.21% of the overall community variation, while the vertical axis explained 15.54% (*p* = 0.001, Figure 2C). The diseased group exhibited clustering with the phage diseased group, indicating similarities in bacterial communities between these two groups. Moreover, there was a relatively comparable distribution of bacterial composition between the healthy group and the phage asymptomatic group. These findings suggest that the application of phage cocktail treatment significantly influenced the composition of endophytic bacterial communities in the kiwifruit branches. The beta diversity analysis revealed substantial alterations in the community structure of branch endophytic bacteria within kiwifruit branches following phage cocktail treatment, resulting in a closer resemblance to the community composition observed in healthy plants rather than diseased ones.

The endophytic bacterial composition of the kiwifruit branches showed significant differences (Figure 2D). At the family level, *Beijerinckiaceae* (16.07%, 7.52%), *Rhizobiaceae* (5.78%, 3.57%), *Sphingomonadaceae* (12.27%, 11.83%), and *Xanthomonadaceae* (14.34%, 17.05%) were found to be dominant in both the healthy group and the phage asymptomatic group, respectively. Moreover, in comparison to the healthy group and the phage asymptomatic group, the diseased group and the phage diseased group demonstrated a substantial enrichment of certain ASVs within the endophytic community of branches, including *Pseudomonadaceae* (29.54%, 17.36%), *Pectobacteriaceae* (7.88%, 35.65%), *Yersiniaceae* (21.94%, 16.40%), and *Enterobacteriaceae* (3.96%, 10.14%), among others.

These findings indicate that treatment with a phage cocktail significantly influences not only diversity, but also the abundance and composition of bacterial communities within kiwifruit branches.

### 3.3. Co-Occurrence Network of Branch Endophytic Bacterial Communities

By employing co-occurrence network analysis, symbiotic associations among ASV bacterial communities in the branches under distinct treatments were successfully discerned. These responsive bacteria predominantly inhabited diverse modules within the endophytic bacterial communities of the branches.

**Figure 3:**
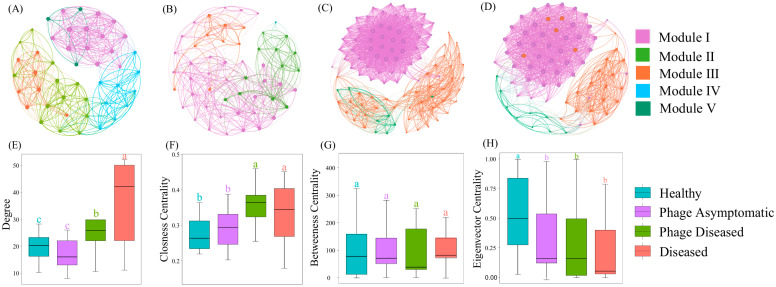
Co-occurrence network patterns of branch endophytic bacterial communities. The co-occurrence network showed the connectivity between the core branch endophytic ASVs of healthy (**A**), phage asymptomatic (**B**), diseased (**C**), and phage diseased (**D**) groups. Comparison between node-level topological features such as degree (**E**), closeness centrality (**F**), betweenness centrality (**G**), and eigenvector centrality (**H**) in each group. Means not sharing the same letter are significantly different by the *t*-test or Wilcoxon test (*p* < 0.05). The areas of different colors represent aggregation modules of branch endophytic bacterial communities; the lines between the nodes represent the correlation between the connected ASVs.

The modules of the healthy group (Figure 3A) and the phage asymptomatic group (Figure 3B) exhibited significant aggregation and overlap, while the diseased group (Figure 3C) and the phage diseased group (Figure 3D) were distinctly separated. Each of the four groups, namely healthy, phage asymptomatic, diseased, and phage diseased, displayed a co-occurrence network with node–edge relationships consisting of 45 nodes and 494 edges, 53 nodes and 440 edges, 75 nodes and 1249 edges, and 103 nodes and 1656 edges, respectively. The modularity values for these groups were calculated to be as follows: healthy 1.656; phage asymptomatic 3.259; diseased 0.801; phage diseased 0.447, respectively, indicating that both the kiwifruit branch endophytic community structure in the healthy group and the phage asymptomatic group demonstrated a more pronounced modular network than the other two groups. By integrating the topological metrics of the co-occurrence network, such as node degree and closeness centrality, we can identify key hub bacteria across the four distinct groups: *Sphingomonadaceae* (the healthy group), *Beijerinckiaceae* (the phage asymptomatic group), *Pseudomonadaceae* (the diseased group), and *Yersiniaceae* (the phage asymptomatic group).

Additionally, the different treatments induced modifications in the topological characteristics of subcommunities (degree, closeness centrality, betweenness centrality, and eigenvector centrality) within distinct ecological clusters in the co-occurrence network. The degree and closeness centrality values were higher for both the diseased group and phage diseased group compared to the healthy group and phage asymptomatic group (Figure 3E,F). No significant differences were observed in betweenness centrality among the four subgroups (Figure 3G). However, the eigenvector centrality in the healthy group was found to be higher than that observed in the other groups (Figure 3H). The phage cocktail treatment exerted a positive impact on the structural integrity and stability of the modular network, thereby confirming that after phage cocktail treatment, the co-occurrence pattern of branch endophytic community structure in kiwifruit branches closely resembled that of the healthy group.

### 3.4. Effect of Phage Cocktail Treatment on Community Assembly

**Figure 4:**
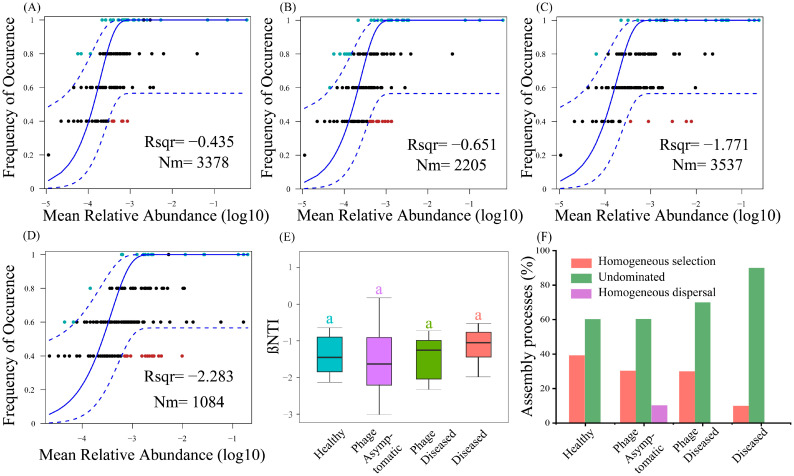
Assembly processes structuring the kiwifruit branch endophytic bacterial community. (**A**–**D**) The relationship between occurrence frequencies and the relative abundance of ASVs for kiwifruit branch endophytic bacterial communities with different treatments. The solid blue line indicates the best fit to the neutral community model, and the dashed blue line indicates 95% confidence intervals. ASVs that occur more or less frequently than predicted by the neutral community model are colored green and red, respectively. R^2^ represents the fit to the model. (**E**) Comparison of βNTI for kiwifruit branch endophytic bacterial communities with different treatments. Means not sharing the same letter are significantly different by the *t*-test or Wilcoxon test (*p* < 0.05). (**F**) The relatively explained ecological processes of kiwifruit branch endophytic bacterial communities based on the values of βNTI and RCbray.

The neutral community model, along with the null model, was employed to investigate the community assemblies of four subcommunities. The neutral model was used to assess the correlation between occurrence frequency and mean relative abundance of ASVs. Interestingly, the analysis revealed that the R^2^ value for all four treatments was negative (Figure 4A–D), suggesting that these groups did not conform to the predictions of the neutral model. Based on the principles of the neutral community model, the findings indicate that deterministic ecological processes are more likely than stochastic ones to shape microbial community formation under phage cocktail treatment.

The mechanics of assembly processes were then elucidated using a null model, which identified the deterministic (|βNTI| ≥ 2) and stochastic (|βNTI| < 2) processes that drive community assemblies (Figure 4E). Moreover, the βNTI values within the range of −2 to +2 accounted for 60% in the healthy group, 70% in the phage asymptomatic group, and 70% in the phage diseased group, while it was 90% in the diseased group. This implies that stochastic processes were more dominantly observed in the community assembly of the diseased communities compared to the healthy communities. Additionally, the proportion of stochastic processes increased with the severity of the disease. βNTI was combined with RC_bray_ to identify the ecological processes governing the assembly (Figure 4F). The phage asymptomatic communities were influenced by homogeneous selection (30%), undominated processes (60%), and homogeneous dispersal (10%). In contrast, the healthy group communities were predominantly shaped by homogeneous selection (40%) and undominated processes (60%). The communities of the phage diseased group were affected by both homogeneous selection (30%) and undominated processes (70%). Homogeneous selection accounted for 10% of the assembly processes, while undominated processes were predominant in the diseased group. As the disease severity increases, there is a progressive increase in the proportion of the microbial community assembly governed by stochastic processes. Conversely, as the kiwifruit becomes healthier, there is a gradual increase in the percentage of deterministic processes. In essence, following phage treatment and subsequent improvement of the disease, the assembly of the kiwifruit branch endophyte community tends towards a deterministic process.

### 3.5. Random Forest Analysis and Diversity Patterns of Abundant and Rare Taxa

**Figure 5:**
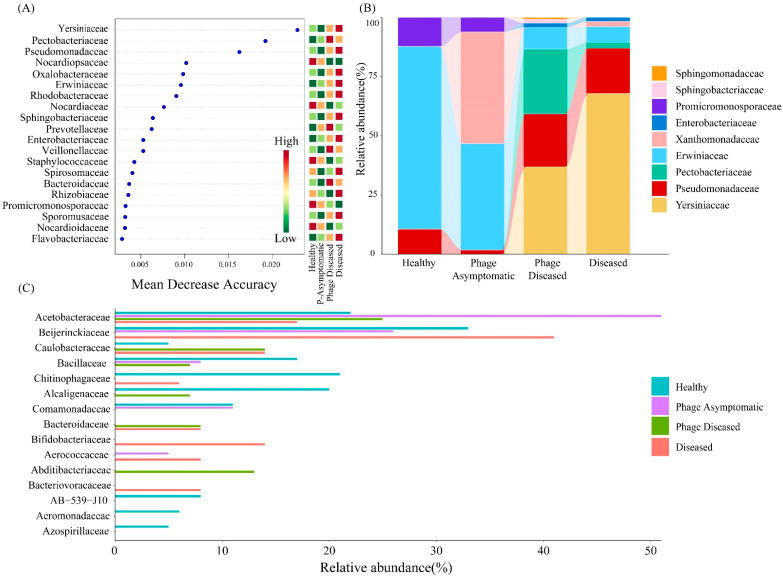
Random forest analysis and the distribution of the rare and abundant communities. (**A**) 20 most important bacteria at the family level that correlated in predicting the different subgroups with high accuracy based on the random forest model. The distribution and relative abundance of the abundant (**B**) and rare (**C**) communities at the family level of the branch endophytic bacterial community in kiwifruit branches.

Using the analytical approach of random forests, a rigorous selection process was employed to identify the family with the highest predictive accuracy for sample subgroups. The comprehensive analysis using the random forest methodology revealed that *Yersiniaceae*, *Pectobacteriaceae*, and *Pseudomonadaceae* emerged as the top three families potentially implicated in canker disease development within kiwifruit branches (Figure 5A).

The bacterial community was divided into three taxonomic groups based on a relative abundance threshold of 0.5% for abundant taxa and 0.01% for rare taxa, with the intermediate taxa representing the middle group. It is noteworthy that there were significant differences in the composition of abundant taxa in the kiwifruit branches (Figure 5B). The dominant taxa in the diseased group were primarily *Yersiniaceae* and *Pseudomonadaceae*. Additionally, the abundant taxa components of the phage diseased group shared similarities with those of the diseased group, but also exhibited the additional presence of *Pectobacteriaceae*. Similarly to the healthy group, the abundant taxa in the phage asymptomatic group included *Promicromonosporaceae*, *Erwiniaceae*, and *Xanthomonadaceae*. A similar abundant taxa structure was observed in both the diseased group and the phage diseased group, while the healthy group and the phage asymptomatic group exhibited more comparable abundant taxa structures. Notably, these findings indicate that the administration of the phage cocktail significantly reduced the abundance of the family *Pseudomonadaceae* containing pathogen *P. syringae* pv. *actinidiae* in the abundant taxa, resulting in lower abundance levels in the phage asymptomatic group. Additionally, the phage cocktail significantly reduced the abundance of *Yersiniaceae* in the rich taxa of the phage asymptomatic group, increasing the abundance from non-rich taxa to 36.8% and from rich taxa to 67.8% compared to the phage diseased and diseased groups. Phage cocktail also increased the abundance of *Pectobacteriaceae* in the phage diseased group, elevating it from non-rich taxa to 27.3%. Notably, *Xanthomonadaceae* demonstrated substantial levels of abundance (47.2%) among the abundant taxa within the phage asymptomatic group.

The rare taxa (Figure 5C) exhibited a higher taxonomic diversity compared to the abundant taxa. Specifically, the rare taxa of all four groups comprised 15 families, including *Acetobacteraceae*, *Beijerinckiaceae*, *Caulobacteraceae*, *Bacillaceae*, *Chitinophagaceae*, and *Alcaligenaceae*, etc. Notably, *Acetobacteraceae* was consistently present in all four groups of rare taxa with substantial abundance levels, whereas AB−539−J10, *Aeromonadaceae*, and *Azospirillaceae* were exclusively found in the rare taxa of the healthy group. *Bacillaceae*, as a rare taxon, was present in all groups except the diseased group. *Bacteriovoracaceae* only existed in the rare taxa of the diseased group. Additionally, *Abditibacteriaceae* was found exclusively in the rare taxa composition of the phage diseased group. Notably, both healthy and phage asymptomatic groups contained *Comamonadaceae* as a unique rare taxon, while it was absent in other groups. Furthermore, *Bacteroidaceae* existed as a specific rare taxon for both the phage diseased and diseased groups.

In summary, the family exhibiting the highest prediction accuracy was identified through random forest analysis. The top three families associated with the development of canker disease were determined to be *Yersiniaceae*, *Pectobacteriaceae*, and *Pseudomonadaceae*. A similar conclusion emerged from the analysis of abundant taxa; specifically, *Yersiniaceae* and *Pseudomonadaceae* were prevalent in both the diseased group and the phage diseased group as abundant taxa with high abundance. In contrast, the composition of abundant taxa in the healthy group and phage asymptomatic group showed greater similarity. Furthermore, an analysis of rare taxa indicated that the phage cocktail could alter their abundance, thereby impacting the endophytic bacterial community within kiwifruit branches.

## 4. Discussion

The bacterial canker caused by *P. syringae* pv. *actinidiae* has been widely acknowledged as the most devastating disease in kiwifruit production. Numerous countries have reported substantial losses attributed to this pathogen-induced ailment [7,8], and the development of eco-friendly biocontrol strategies against *P. syringae* pv. *actinidiae* infection is urgently needed. This study aimed to assess the efficacy of phage cocktails in managing kiwifruit canker in the field, as well as investigate alterations in community structure and assembly mechanisms within kiwifruit branch endophytic bacterial communities influenced by the application of phage cocktails.

Our field trial demonstrated a significant reduction in the incidence of kiwifruit canker disease after phage cocktail treatment (Table 1), exhibiting a high efficacy rate under field conditions, consistent with previously reported findings [17,42]. Compared to healthy branches, kiwifruit branches affected by canker disease exhibited significantly reduced levels of alpha diversity in branch endophytic bacteria. The invasion of or interactions with other bacteria caused by *P. syringae* pv. *actinidiae* could potentially alter the structure and diminish the diversity of branch endophytic communities [43]. Compared to the diseased group and the phage diseased group, both the healthy group and the phage asymptomatic group exhibited significantly higher Shannon index and Pielou index values. The PCoA distribution of the healthy and phage asymptomatic groups was distinctly separated from that of the diseased and phage diseased groups. The Bray–Curtis distances of the samples from the diseased group and the phage diseased group were closer, while the samples from the healthy group were closer to the phage asymptomatic group. This observation suggests a higher degree of similarity in terms of diversity and composition between the diseased group and the phage diseased group, as well as between the healthy group and the phage asymptomatic group. These findings showed that treatment with the phage cocktail significantly impacted both alpha and beta diversity, leading to an increase in richness within kiwifruit branch endophytic bacterial communities compared to the diseased and phage diseased groups. Furthermore, the phage cocktail treatment restored community diversity and brought microbial composition closer to that of uninfected states.

The relative abundance of the family *Pseudomonadaceae* in the diseased group, phage diseased group, phage asymptomatic group, and healthy group decreased sequentially by 29.54%, 17.36%, 7.5%, and 5.96%, respectively. It is evident that the application of the phage cocktail significantly reduced the relative abundance of *Pseudomonadaceae*. *P. syringae* pv. *actinidiae* along with its diverse pathogenic biovars, which are known to cause damage to a wide range of host crops, representing just one example among numerous conditionally pathogenic plant species belonging to the family *Pseudomonadaceae* [44]. The presence of *Pectobacteriaceae* was barely observed in the healthy and phage healthy groups. Some species belonging to the soft rot *Pectobacteriaceae* (SRP) are some of the most devastating phytopathogens. They degrade plant tissues by producing an arsenal of plant cell wall degrading enzymes [45]. The abundance levels of *Sphingomonadaceae* and *Xanthomonadaceae* remained consistently high in both the healthy group and phage asymptomatic group. The former has the potential to enhance phytoremediation through symbiotic interactions with fungi or other bacteria, such as promoting biosorption, facilitating active efflux transport, and reducing toxicity [46,47]. Numerous *Sphingomonas* spp. have demonstrated their potential in promoting plant growth and enhancing plant resistance, attributed to their nitrogen fixation, phosphate solubilization, and production of plant growth hormones [48,49]. The enrichment of microorganisms from this family in the phage cocktail group may contribute to the restoration of cellular tissues damaged by pathogens, thereby improving the microecology of kiwifruit branch endophytic communities and enhancing community diversity and stability.

The co-occurrence network primarily reflects the co-existence of species within an environment, and plays a pivotal role in elucidating species interactions within a specific ecological setting. Network topological characteristics, including modularity, degree, closeness centrality, betweenness centrality, and eigenvector centrality, can be employed to comprehend the structural stability and complexity of ecological communities. The co-occurrence network analysis revealed that the diseased and phage diseased groups exhibited a significantly higher number of nodes and edges compared to the healthy and phage asymptomatic groups, with the phage diseased group demonstrating the highest count of nodes and edges. Moreover, both the degree and closeness centrality values were found to be higher in the diseased group and phage diseased group compared to the healthy group and phage asymptomatic group. The possible reason for this is that the application of the phage cocktail induced alterations in the structure of kiwifruit branch endophytic communities, thereby triggering interactions between phages and their hosts. Consequently, the process of community restructuring resulted in the formation of a more intricate modular network with enhanced interconnections among nodes. Stronger interconnections between nodes indicate a more stable network, reflecting the improvement in the structural characteristics of the microbial co-occurrence network due to phage treatment. This enhancement facilitates a transition towards a healthier state and bolsters the network’s resistance to disease.

Compared to other treatments, the healthy group exhibited a higher eigenvector centrality, which can be attributed to its greater diversity and richness. This state of health also ensured that network features were maintained at elevated levels. In order to identify factors shaping community composition, it is crucial to investigate the relative significance of deterministic and stochastic processes in community assembly [27]. However, it was agreed that the neutral model alone should not be used to determine the relative significance of ecological processes on community assembly [50]. Therefore, in this study, the neutral model was integrated with the null model. Our findings indicate that the assembly processes of the four subgroups of communities deviate from the predictions of the neutral model, suggesting a stronger influence of deterministic processes on community assembly. By employing the null model theory, it was observed that stochastic processes predominantly governed community assembly in the diseased group compared to the healthy group. Furthermore, as disease severity increased, undominated processes (representing stochastic processes) steadily accounted for up to 90% of community assembly in the diseased group.

The application of phage cocktails may account for the relatively high proportion of deterministic processes in community assembly among both the healthy and phage asymptomatic groups. While communities with homogeneous selection as their primary assembly process have been found to exhibit narrower niche breadth, they are also less tolerant to environmental disturbances [22,51,52]. This is one of the potential reasons why certain kiwifruit plants treated with phage cocktails remain diseased, as phages repair the branch endophytic communities, rendering the microbial community more vulnerable to environmental disturbances and resulting in incomplete disease recovery. However, further analysis is required to investigate the extent of the effects of homogeneous selection during the community assembly process in the healthy group.

The random forest analysis revealed that *Yersiniaceae*, *Pectobacteriaceae*, and *Pseudomonadaceae* were the most precise predictors of subgroups. These three families exhibited the highest contribution in both diseased and phage diseased groups, indicating their potential significance in canker disease development. The results of the abundant taxa of the disease group and the phage diseased group were similar to those of the random forest analysis, mainly containing *Yersiniaceae*, *Pseudomonadaceae*, *Pectobacteriaceae*, *Erwiniaceae*, etc. The *Yersiniaceae* are a family of Gram-negative bacteria that includes some familiar pathogens. For example, the type genus *Yersinia* includes *Yersinia pestis*, the causative agent of plague [53]. Multiple infections of some members of *Yersiniaceae* with *P. syringae* pv. *actinidia* may exacerbate disease progression during the development of kiwifruit canker disease.

The common rare taxa present only in the healthy group and the phage asymptomatic group is the *Comamonadaceae*, a family of the *Betaproteobacteria*. Some members of *Comamonadaceae* have an aerobic denitrification function that promotes biological nitrogen removal in the microbial community. They can also degrade pollutants in the microbial environment and promote ecological remediation [54]. This may indicate a link between the enrichment of *Comamonadaceae* and healthy maintenance.

This study demonstrates that the phage cocktail significantly contributes to disease reduction by selectively targeting pathogens with high phage resistance and limiting their growth potential in branches, thereby maintaining pathogen density at a specific threshold while reducing disease incidence [19]. Studies have shown that the effects of phage biocontrol are mediated through the lysis of pathogens by phages and various evolutionary mechanisms. Specifically, disease reduction is linked to selective pressures against highly resistant yet slow-growing pathogens. Additionally, it has been observed that certain bacterial taxa, whose abundance positively correlates with the phage treatment, exhibit a significant degree of antagonism against pathogens via resource competition or interference competition [42]. Our study also revealed that the phage cocktail indirectly influenced the diversity, community composition, co-occurrence networks, and community assembly processes of branch endophytic microbial communities. It is hypothesized that these phage-driven alterations in the branch endophytic microbiome community may promote the presence of antagonistic bacteria among resident microorganisms and restore community diversity to a healthy state. Future investigations involving functional analyses and culture studies are necessary to fully comprehend the role of enriched microorganisms. To draw more robust conclusions, it is essential to further isolate and cultivate the potentially beneficial bacteria that have been enriched and explore their effects on disease resistance and growth enhancement.

## 5. Conclusions

In this study, we investigated the biocontrol efficacy of the phage cocktail against kiwifruit canker disease in field conditions and examined the composition and assembly processes of kiwifruit branch endophyte communities across different groups. The application of the phage cocktail exhibited remarkable effectiveness in managing canker disease by significantly reducing bacterial incidence, modulating the structure of branch endophytic bacterial communities, regulating community modular networks, and promoting deterministic assembly processes. Consequently, it facilitated the convergence of kiwifruits’ branch bacterial community towards that observed in healthy plants. These findings provide valuable technical support for field-based control strategies against kiwifruit bacterial canker and contribute to enriching biocontrol approaches utilizing phage cocktails.

## Figures and Tables

**Table 1:** Effects of phage cocktail on bacterial canker of kiwifruit in field.

Year of Field Trial	Groups	Diseased Number	Effective Rate (%)	Mean of Disease Index *
2023	Phage cocktail	42	46.8%	1.81 ± 0.85 ^b^
	Diseased	79	−	2.82 ± 1.17 ^a^
2024	Phage cocktail	37	50.7%	1.51 ± 0.55 ^b^
	Diseased	75	−	3.39 ± 1.15 ^a^

−: No effective rate can be calculated. Lowercase letters represent a significant difference between 2 groups (*p* < 0.001). * Only the kiwifruit plants with bacterial canker symptoms were counted.

## Data Availability

The raw sequence data for the 16S rRNA gene of all samples were submitted to the NCBI Sequence Read Archive database with the accession no. PRJNA1037357 (https://www.ncbi.nlm.nih.gov/bioproject/PRJNA1037357/ (accessed on 9 November 2023)).

## Supplementary Materials

The following supporting information can be downloaded at: https://www.mdpi.com/article/10.3390/microorganisms13010104/s1, Table S1: the resequencing statistics; Table S2: ASV details.
