# Supplementary material for: Phage Cocktail Alleviates Bacterial Canker of Kiwifruit by Modulating Bacterial Community Structure in Field Trial
Source: Microorganisms. 2025 Jan 7;13(1):104. doi: 10.3390/microorganisms13010104 (PMC11767704; doi:10.3390/microorganisms13010104)
Supplement: Supplementary file 1 [file microorganisms-13-00104-s001.zip › Supplementary Table S1 The resequencing statistics.pdf]

Supplementary materials

Supplementary Table S1 The resequencing statistics

Sample		Raw tags	Clean tags
healthy	H1	61540	46891
	H2	64207	50758
	H3	69514	58511
	H4	68571	57495
	H5	59923	33979
phage asymptomatic	PA1	71628	63578
	PA 2	72130	63772
	PA 3	71671	63402
	PA 4	71649	59356
	PA 5	68533	54956
phage diseased	PD1	66118	55360
	PD 2	68121	61195
	PD 3	67625	59636
	PD 4	75419	67891
	PD 5	65106	52861
diseased	D1	56708	52672
	D 2	46850	40224
	D 3	63097	61836
	D 4	58060	48626
	D 5	58857	57748
